# Transient Co-Expression of Post-Transcriptional Gene Silencing Suppressors for Increased *in Planta* Expression of a Recombinant Anthrax Receptor Fusion Protein

**DOI:** 10.3390/ijms12084975

**Published:** 2011-08-05

**Authors:** Lucas Arzola, Junxing Chen, Kittipong Rattanaporn, James M. Maclean, Karen A. McDonald

**Affiliations:** 1 Department of Chemical Engineering and Materials Science, University of California, Davis, One Shields Ave, Davis, CA 95616, USA; E-Mails: larzola@ucdavis.edu (L.A.); cicchen@ucdavis.edu (J.C.); krattanaporn@ucdavis.edu (K.R.); 2 Planet Biotechnology Inc., 25571 Clawiter Road, Hayward, CA 94545, USA; E-Mail: jmaclean@planetbiotechnology.com

**Keywords:** molecular farming, transient, gene silencing suppressors, *Nicotiana benthamiana*, anthrax, fusion protein

## Abstract

Potential epidemics of infectious diseases and the constant threat of bioterrorism demand rapid, scalable, and cost-efficient manufacturing of therapeutic proteins. Molecular farming of tobacco plants provides an alternative for the recombinant production of therapeutics. We have developed a transient production platform that uses *Agrobacterium* infiltration of *Nicotiana benthamiana* plants to express a novel anthrax receptor decoy protein (immunoadhesin), CMG2-Fc. This chimeric fusion protein, designed to protect against the deadly anthrax toxins, is composed of the von Willebrand factor A (VWA) domain of human capillary morphogenesis 2 (CMG2), an effective anthrax toxin receptor, and the Fc region of human immunoglobulin G (IgG). We evaluated, in *N. benthamiana* intact plants and detached leaves, the expression of CMG2-Fc under the control of the constitutive CaMV 35S promoter, and the co-expression of CMG2-Fc with nine different viral suppressors of post-transcriptional gene silencing (PTGS): p1, p10, p19, p21, p24, p25, p38, 2b, and HCPro. Overall, transient CMG2-Fc expression was higher on intact plants than detached leaves. Maximum expression was observed with p1 co-expression at 3.5 days post-infiltration (DPI), with a level of 0.56 g CMG2-Fc per kg of leaf fresh weight and 1.5% of the total soluble protein, a ten-fold increase in expression when compared to absence of suppression. Co-expression with the p25 PTGS suppressor also significantly increased the CMG2-Fc expression level after just 3.5 DPI.

## 1. Introduction

Epidemics of recent emerging infectious diseases such as avian influenza, or more recently the H1N1 pandemic, demand cost-efficient and scalable manufacturing technologies that can rapidly deliver effective therapeutics into the clinical setting. The threat of bioterrorism also makes evident the need for facilitating the production of therapeutic proteins for the mitigation of potential outbreaks. Current manufacturing technologies for therapeutics, such as mammalian cell culture or chicken eggs, are ill-equipped to supply the demand such an outbreak would create, and are simply not able to produce therapeutics on a large scale in a short amount of time [1,2]. Advances in the field of molecular farming have positioned plant-made pharmaceuticals as a viable option in the market [3–5]. Using plants as production hosts for recombinant proteins is an attractive option because it offers important features such as cost-effectiveness, scalability, and safety. Plant-based production can replace traditional infrastructure required in the upstream portion of manufacturing facilities [6], and several acres of biomass would be sufficient to generate millions of protein doses over the course of a year [7]. Protein expression in plants is also adequate for production of human therapeutics, because plants do not harbor or propagate human viruses or pathogens, while still being able to perform protein post-translational modifications [8].

Expression of plant-made proteins in a transient fashion–DNA is transcribed and translated without integration into the plant host genome—instead of production in transgenic plants, has recently become a particularly interesting approach for biomanufacturing. *Agrobacterium tumefaciens* mediated transformation, utilizing agroinfiltration, can be used to rapidly enable the transient expression of a recombinant protein of interest. Transient expression offers several advantages such as: short timelines for protein production [9–11]; increased yields due to a burst of gene expression [12]; the capability of co-expressing multiple recombinant proteins simultaneously [13]; and the ability to use available plant biomass for large scale protein manufacturing [14]. Large scale transient expression is a contained process that does not generate transgenic plants, and therefore, it is a preferable approach to plant-made protein manufacturing because it eliminates environmental concerns about transgene release and reduces regulatory concerns [7]. Biotech companies are already researching the use of transient expression for production of therapeutic proteins, mainly for production of influenza subunit vaccines. Several of them have products for which they have performed immunization challenges in animals [15,16], shown pharmaceutical-grade production [17], or are currently undergoing clinical trials [1].

Since the 2001 anthrax mailings, anthrax has been designated as the number one biological warfare threat to the United States. Bioterrorists can isolate and purify the anthrax spores from *Bacillus anthracis*, which can then be grown *in vitro* and used as a biological weapon [18]. Anthrax infection in humans leads to the release of toxins, which are fatal if not properly treated [19]. The anthrax toxins are a complex of three proteins that work cooperatively. The first, Protective Antigen (PA), binds directly to the effective anthrax cell receptor on the cell, capillary morphogenesis protein 2 (CMG2) [20]. The other two proteins, edema factor (EF) and lethal factor (LF), bind to PA and form edema toxin and lethal toxin. Inside the cell, both toxins trigger a cascade of events that cause disruption of signal transduction pathways and culminate in necrosis [21].

Inhibiting the binding of PA to the CMG2 receptor has been a major focus of researchers seeking to develop an effective treatment for anthrax. Antitoxins based on receptor decoys are a promising alternative, because it is difficult to engineer a PA that can escape binding to the CMG2 decoy without compromising its binding to the endogenous CMG2 receptor. A soluble form of CMG2 has been shown to inhibit intoxication of cells expressing endogenous toxin receptors [20]. Further research has been performed in the creation of CMG2-Fc fusion proteins, because the immunoglobulin fragment confers CMG2 the half-life, multivalency, and potency of an antibody. The CMG2-Fc target protein of this research project is one such chimeric antibody, or immunoadhesin, that was generated by combining the Fc region of human IgG and the VWA domain of the CMG2 anthrax receptor, and has already been shown to be effective against anthrax lethal toxin *in vitro* [22].

In this research project, we transiently produce CMG2-Fc in *Nicotiana benthamiana* under the control of the CaMV 35S constitutive promoter, and test the effect of suppressing post-transcriptional gene silencing (PTGS) on the protein production kinetics. PTGS is a natural defense mechanism for plants against viruses and pathogens [23]. It is a virus-induced mechanism that relies on RNA interference, through which double stranded RNAs trigger silencing in the plant. Plant viruses evolved to counteract this antiviral mechanism, and in the process developed to encode proteins known to suppress PTGS in plants [24]. In fact, most plant viruses are expected to carry suppressors of gene silencing, presenting a variety of sequences and modes of action over the RNA interference pathway [25]. PTGS suppression has been shown to increase yields of transiently expressed reporter proteins in plants [26], and we wanted to explore this mechanism as it pertains to increasing the expression of the foreign CMG2-Fc in the plant. In this experiment, we tested the co-expression of PBI-220 with nine different viral proteins, all reported in the literature to be suppressors of PTGS (Table 1).

The goal of this research was to test the effect of the PTGS suppressors mentioned above on the expression level of CMG2-Fc in *Nicotiana benthamiana*. Our aim was to perform the delivery of the *Agrobacterium* containing the genes for the protein of interest and the suppressor proteins into the plants in a way that translates to a scaled-up industrial process. For this reason, we selected vacuum infiltration as the method for *Agrobacterium* delivery.

This experiment also tested two distinct transient expression approaches, because of their implications in the scalability and cost-effectiveness of protein production in plants. In the intact plant approach, we agroinfiltrated whole *N. benthamiana* plants and incubated them in a greenhouse for the duration of the experiment. In the detached leaf approach, we harvested the leaves immediately before agroinfiltration, and incubated them in the dark inside plastic containers with controlled temperature and humidity for the duration of the experiment.

## 2. Results and Discussion

### 2.1. Effect of PTGS Suppressor Co-Expression on CMG2-Fc Expression Level in Intact Plants

**Figure 1.**
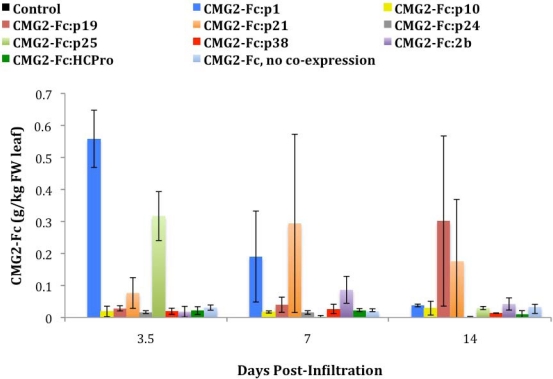
ELISA analysis of transiently expressed CMG2-Fc on a leaf FW basis in intact plants in which the p1, p10, p19, p21, p24, p25, p38, 2b, and HCPro PTGS suppressors were co-expressed. An infiltration of CMG2-Fc without PTGS suppressor co-expression was also performed to have a baseline for comparison. CMG2-Fc expression levels are the average of triplicate *N. benthamiana* leaves exposed to the same condition. Error bars are based on the propagation of errors calculated from triplicate assays performed on samples of triplicate *N. benthamiana* leaves from the intact plants.

ELISA analysis was used to quantify the CMG2-Fc protein that was transiently expressed within the leaves of the *N. benthamiana* intact plants (Figure 1). Bradford assays were also performed to quantify the levels of total soluble protein (TSP) expressed within the *N. benthamiana* intact plants. Values obtained from the Bradford assays were combined with ELISA data to determine the CMG2-Fc expression level as a percentage of the TSP. The effect of co-expressing certain suppressor proteins on the CMG2-Fc expression level was dramatic, when compared to the baseline agroinfiltration in which CMG2-Fc was tested without co-expression of the PTGS suppressor. Transient expression in intact plants was observed to be higher with p1 co-expression at 3.5 days, resulting in a maximum average production of 0.56 g PBI-220 per kg of leaf fresh weight (FW) and 1.5% of the TSP. With p1, the CMG2-Fc expression level increased ten-fold when compared to the absence of suppressor co-expression, and was statistically different at a 95% confidence level. Co-expression with the p25 PTGS suppressor was also found to be significantly different from the results obtained in the absence of suppressor at 3.5 days. The rest of the suppressor proteins tested had no demonstrated effect on CMG2-Fc yield, since they showed comparable expression levels that did not statistically differ from CMG2-Fc without co-expression. As expected, the negative control consisting of wild type plants infiltrated only with buffer that did not contain *Agrobacterium* did not show CMG2-Fc expression.

**Figure 2.**
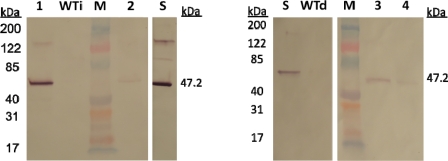
Western Blot analysis of CMG2-Fc protein from intact *N. benthamiana* plants reduced with 90 mM dithiothreitol (DTT). Samples shown below were obtained from plants harvested at either 3.5 or 14 days post-infiltration (DPI). Lanes 1 and 2 show CMG2-Fc expression on intact plants: CMG2-Fc co-expressed with gene silencing suppressor p1 at 3.5 DPI (lane 1); CMG2-Fc without gene silencing suppressor co-expression at 3.5 DPI (lane 2). Lanes 3 and 4 show CMG2-Fc expression on detached leaves: CMG2-Fc co-expressed with gene silencing suppressor p19 at 14 DPI (lane 3); CMG2-Fc without gene silencing suppressor co-expression at 14 DPI (lane 4). Lane M: molecular weight marker; lane WTi: control infiltration of an intact plant (without *Agrobacterium*); lane WTd: control infiltration of a detached leaf (without *Agrobacterium*); lane S: purified CMG2-Fc protein standard (2000 ng). Purified CMG2-Fc was used as a positive control to allow comparisons between the relative band intensities. Approximate sizes (kDa) of proteins are shown.

CMG2-Fc expression in intact plants is also evident based on the Western Blot analysis (Figure 2). A major immunoblot band migrated as expected to approximately 47.2 kDa. Dimeric and trimeric bands can also be observed at approximately 94.4 kDa and 141.6 kDa, respectively. It is expected that full-length CMG2-Fc will run a bit higher on a SDS-PAGE because the protein has one *N*-glycosylation site. The values measured with ELISA allowed us to predict the amount of CMG2-Fc loaded in each lane for the Western analysis (Table 2). In order to compare the relative band intensities, 2000 ng of purified CMG2-Fc were loaded as a positive control, corresponding to an amount of 1 g/kg FW leaf. Western Blot analysis confirmed that CMG2-Fc expression at 3.5 days was strong when co-expressed with p1. The negative control infiltration did not result in any immunoreactive bands.

### 2.2. Effect of PTGS Suppressor Co-Expression on CMG2-Fc Expression Level in Detached Leaves

**Figure 3.**
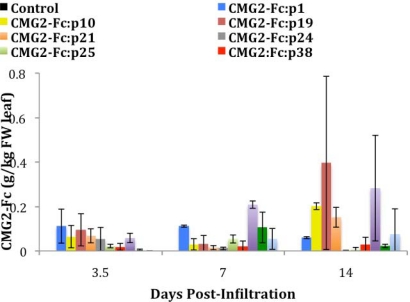
ELISA analysis of transiently expressed CMG2-Fc on a leaf FW basis in detached leaves co-expressed with p1, p10, p19, p21, p24, p25, p38, 2b, and HCPro PTGS suppressors. An infiltration of CMG2-Fc without PTGS suppressor co-expression was performed to have a baseline for comparison. CMG2-Fc expression levels are the average of triplicate *N. benthamiana* leaves exposed to the same condition. Error bars are based on the propagation of errors calculated from triplicate assays performed on samples of triplicate *N. benthamiana* leaves.

CMG2-Fc was also quantified from the samples obtained from *N. benthamiana* detached leaves using ELISA (Figure 3). In this case, the best performer was p19 co-expression at 14 DPI, which showed an average expression level of 0.39 g CMG2-Fc per kg of leaf FW, corresponding to 2% of the TSP. This is a five-fold increase when compared to the absence of suppressor co-expression, but due to the variability observed, was not found to be significantly different using a *p*-value of 0.05. The 2b suppressor protein slightly increased the CMG2-Fc expression level, although not at a statistically significant level. The negative control infiltration showed no CMG2-Fc expression as expected.

Western Blot analysis proved that CMG2-Fc was also expressed correctly in the *N. benthamiana* detached leaves (Figure 2). Immunoblot bands can be observed exclusively at the 47.2 kDa level. Western Blot also confirms that the protein of interest was expressed under p19 co-expression, and that CMG2-Fc was present in the detached leaves even at 14 DPI. We also detected CMG2-Fc expression at 3.5, 7, and 14 DPI in both intact plants and detached leaves for several of the other suppressor proteins tested (data not shown). No bands appeared on the negative control lane.

These results show that suppressing the post-transcriptional gene silencing mechanism in *N. benthamiana* increased the expression level of a recombinant protein expressed transiently under the control of the CaMV 35S promoter. While co-expression of certain suppressor proteins enhanced CMG2-Fc expression, other suppressor proteins had no apparent positive effect on CMG2-Fc accumulation. There have been reports in the literature in which PTGS suppression was not able to increase the expression level of a plant-made recombinant protein under the control of the CaMV 35S promoter [36]. Therefore, it is important to complete the type of screening analysis performed in this project for each new plant system and/or target product of interest, in order to determine the conditions that optimize recombinant protein yields.

Wycoff *et al*. [37] recently reported a high expression level of CMG2-Fc in *Nicotiana benthamiana*. Optimized expression enabled these researchers to obtain 0.73 g of CMG2-Fc per kg of leaf FW using the p19 gene silencing suppressor, the highest level reported for this fusion protein. Wycoff *et al*. evaluated different combinations of CMG2 and IgG1 to develop an improved CMG2-Fc construct. The CMG2-Fc construct utilized in the project reported here is not the current optimized construct, hence the lower expression of 0.56 g of CMG2-Fc per kg of leaf FW, even with gene silencing suppressor co-expression. Regardless, this project provides comparative data on PTGS suppressor co-expression for production of CMG2-Fc, which complements the construct optimization work of Wycoff *et al*. Both approaches will be important for further optimization of CMG2 production towards expression levels on the order of gram of protein per kilogram of *N. benthamiana* leaf tissue.

No common trends were identified for the CMG2-Fc expression kinetics using different gene silencing suppressors, which may be due to the diversity of the suppressor proteins tested. Different plant viruses evolved independently to counter the antiviral defense of the plants, as evidenced by their different modes of action against the gene silencing phenomenon. The fact that the suppressor proteins tested in this study target different steps of the RNA interference mechanism may explain the differences observed in the kinetic data. Three of the best performers in this experiment (p19, p21, and 2b) bind dsRNA, which may indicate that pathway specific effects are in play. The p19 protein from tomato bushy stunt virus is a strong silencing suppressor protein and has a unique mechanism by selectively binding to the 21-nt and 22-nt classes of siRNA. The p21 protein from Yellow Beet Virus and the 2b protein from Cucumber Mosaic Virus are both silencing suppressor proteins that can bind siRNA and longer dsRNA without a preference for 21-nt siRNAs. Meanwhile, the p1 protein from Rice Yellow Mottle Virus, which increased CMG2-Fc expression the most when compared to absence of suppressor, has a different mechanism. It inhibits the accumulation of the 24-nt siRNA, but has a less pronounced effect on the accumulation of the 21-nt siRNA [38].

Another possible explanation for the differences in the data may be the vectors used in this experiment. pBIN (11.8 kb) and pCB302 (3.5 kb) are two of the most widely used vectors for *Agrobacterium*-mediated plant transformation in plants. There is sequence homology between both vectors due to the fact that pCB302 was developed by removing base pairs that were deemed non-essential on pBIN. pCB302 retains all essential elements of pBIN, and its smaller size makes it more versatile for cloning [39]. Still, using two different vectors to drive the expression of the suppressors may have had an effect on the expression level of the suppressors, and therefore, on the accumulation of CMG2-Fc. In fact, the highest expression levels obtained in the experiment were from suppressors that were inserted on pBIN (p1, p19, p25, and 2b), so it is possible that pBIN could be a more suitable vector for this particular system.

During the agroinfection process in plants, the antagonistic forces of plant pathogen invasion and defense are governed by a dynamic set of biological components. There are two possibilities that may explain the observation that the expression level of a CaMV 35S driven recombinant protein is being increased by suppression of PTGS. The first possibility could be that *Agrobacterium* may be activating the gene silencing mechanism in the plant. *Agrobacterium*, acting as the plant pathogen in *Nicotiana benthamiana*, may be producing protein effectors that elicit the production of small RNAs in the plant. The plant might be deploying a gene silencing approach to counter the *Agrobacterium* infection, and co-expression of gene silencing suppressors may be counteracting the gene silencing response. Previous studies have shown that bacterial effectors can act as suppressors [40] of gene silencing, but no instance has been reported in which these proteins can act as gene silencing activators. Interestingly, a dual function of activation and suppression of gene silencing has been recently found for a viral protein previously identified as a gene silencing suppressor [41]. It has been established that infiltration with *Agrobacterium* elicits a distinct host response in plants, but the mechanisms are still not well understood [42].

The second possibility for explaining our observations is that the overexpression of a foreign gene is inducing the PTGS mechanism, through a quantitative effect exerted by the RNA derived from the transgene. PTGS has been identified as a plant defense mechanism for aberrant gene expression following agroinfection, such as production of excessive RNA transcripts over a threshold level [43,44]. Several groups have reported that overexpressing a transgene over a certain level causes transgene silencing through activation of PTGS [45–47], including when occurring under CaMV 35S promoter control [48,49]. This evidence supports a theory that PTGS occurs due to the elevated CaMV 35S driven CMG2-Fc expression, regardless of the absence of viral RNAs. Under these conditions, CMG2-Fc expression can be increased when co-expressed with gene silencing suppressors.

It is clear that CMG2-Fc levels change over time, so the same *N. benthamiana* leaves were sampled along all time points post-infiltration to study the kinetics of production. For some of the transient expression experiments, a high degree of variability was observed between leaves, which is consistent with what others have observed [50]. This variability arose from the differences between the sample replicates (leaves are sampled from different plants), and not from the assays used for protein quantification. It is possible that variations in protein expression are due to the application technique, the sampling method, or protein stability issues. Due to position effects within the leaves, there is inherent variability in the expression of recombinant proteins in plants. A study by Sheludko *et al*. [51] evaluated sources of variability in the transient expression of a recombinant protein in *Nicotiana* plants, and determined that factors such as the age of the plant and the location of the leaf within the plant can cause differences in expression. Applying *Agrobacterium* through syringe infiltration may reduce variability compared with vacuum infiltration, although it is not a method that could be used at large scale. Variability may be reduced by sampling the entire leaf and measuring protein expression from that extract, but this approach would preclude the possibility of examining the expression kinetics. Finally, denaturation, degradation, unfolding, or cleavage of the protein could also have an impact on how much of the protein is measured, as reported for other proteins expressed transiently [52].

## 3. Experimental Section

### 3.1. Cloning of the CMG2-Fc and Gene Silencing Suppressor Constructs for Agrobacterium Infiltration

A DNA fragment encoding a chimeric human IgG1/IgG2 Fc region (include the upper and middle hinge sequences of human IgG2, followed by the lower hinge, CH2, and CH3 regions of IgG1) was codon-optimized for *Nicotiana tabacum* expression. Nucleotides encoding the peptide sequence SEKDEL were appended onto the 3′ end to enable accumulation in the plant’s endoplasmic reticulum. This Fc2/1-KDEL sequence was cloned into pMSP-CMG2-Fc_G_ [37] between the SacI and EcoRI sites, replacing the Fc_G_ sequence (non-chimeric IgG1 Fc). An AscI digested fragment from pMSP-CMG2-Fc2/1-KDEL was sub-cloned into the *Agrobacterium* binary vector pBIN+ocs [53], forming pBIN-CMG2-Fc2/1-KDEL. The final expression cassette was comprised of the CaMV 35S promoter, a short 5′ untranslated region from the *Nicotiana sylvestris* psaDb gene (GenBank E12115), the signal peptide of the 2S albumin storage protein of *Arabidopsis thaliana* (GenBank P15458) [54], *Nicotiana* spp. codon-optimized CMG2 gene (amino acids 34 to 220, GenBank AY233452, Fc2/1-KDEL (described above) and the agrocinopine synthase (ags) terminator (GenBank EU181145). The resultant CMG2-Fc2/1-KDEL protein contains 424 amino acids (excluding the signal peptide) having a subunit molecular mass of 47.2 kDa (dimer of 94.4 kDa). The pBIN-CMG2-Fc2/1-KDEL binary expression vector was transformed into *A. tumefaciens* EHA105 via electroporation [55].

Each of the plant viral suppressor sequences from p1, p19, p25, and 2b were inserted into the pBIN binary vector. Likewise, the DNA sequences of p10, p21, p24, p38, and HcPro were cloned into a mini-binary vector, pCB302 [39]. All of the gene silencing suppressors used in this study were under the control of the CaMV 35S promoter. The binary vectors containing gene silencing suppressor sequences were transformed into *A. tumefaciens* EHA105, with the helper plasmid (pCH32) by electroporation.

### 3.2. Preparation of *Nicotiana benthamiana* Plants

Wild type non-transgenic *N. benthamiana* was grown from seeds planted in soiled-filled seed trays. Seedlings were transplanted into individual 4-inch pots. A week after transplantation, Osmocote fertilizer (Scotts Miracle-Gro Company, Marysville, OH) was added to ensure optimum health throughout the growth cycle. Plants that were 4–6 weeks old were used for agroinfiltration. In the case of detached leaf agroinfiltration, upper leaves from 4–6 weeks old *N. benthamiana* plants were harvested immediately before agroinfiltration. All plants were grown in a greenhouse with a 16-h photoperiod and a temperature range of 18 °C (night time low) to 30 °C (day time high).

### 3.3. Preparation of *Agrobacterium tumefaciens* for Agroinfiltration

*Agrobacterium* cultures were started from −80 °C frozen stocks, and were grown in 5 mL of LB broth. After incubation for 24 h at 28 °C and 250 rpm, the 5 mL cultures were transferred to 250 mL Erlenmeyer flasks containing 125 mL sterile LB media. After incubating for 24 h at 28 °C and 250 rpm, the *Agrobacterium* cells were harvested by centrifugation at 1890 g for 10 min. Cells were resuspended in an infiltration buffer, containing 100 mM acetosyringone (3′,5′-dimethoxy-4′-hydroxyacetophenone) (Aldrich Chemicals, Milwaukee, WI), 1 mL of 1 M 2-(4-morpholino) ethanesulfonic acid (MES) buffer (pH 5.6), and 10 mM magnesium chloride. The cell density (optical density, OD_600_ obtained using a Milton Roy Spectronic 501 spectrophotometer) of the agroinfiltration solution was adjusted to 0.5 absorbance units for processing intact plants. For agroinfiltration of detached leaves, the agroinfiltration solution was adjusted to 1.0 absorbance units. An *Agrobacterium* suspension containing the CMG2-Fc construct was mixed 1:1 with an *Agrobacterium* suspension containing each of the gene silencing suppressor constructs. For the absence of co-expression condition, the suspension was composed solely of *Agrobacterium* encoding CMG2-Fc. Infiltration buffer without *Agrobacterium* was used as a negative control for CMG2-Fc expression. All infiltration solutions were incubated for three h in the dark at room temperature. Directly before performing the agroinfiltration, the surfactant Silwet L-77 (GE Silicones, Friendly, WV) was added at a final concentration of 0.01% to reduce the surface tension of the solution and facilitate the agroinfiltration.

### 3.4. Agroinfiltration

Whole *N. benthamiana* plants or detached *N. benthamiana* leaves were submerged into the bacterial suspension and subjected to a vacuum of −90 kPa for 1 min, with occasional agitation to release trapped air bubbles. After the vacuum was rapidly released, the plant material was removed from the suspension and allowed to dry. Intact plants were placed in a greenhouse, while detached leaves were placed inside plastic containers prepared as described elsewhere [56].

### 3.5. Sampling and CMG2-Fc Protein Extraction

Leaf discs with a diameter of 14 mm were sampled from agroinfiltrated leaves after 3.5, 7, and 14 days post-infiltration (DPI). Each sample was comprised of two discs obtained from the same leaf. For each condition tested, three individual leaves, from three different *N. benthamiana* plants, were used for analysis. Phosphate buffer saline (PBS) composed of 137 mM NaCl, 2.7 mM KCl, 10.1 mM Na_2_HPO_4_ and 1.8 mM KH_2_PO_4_ (pH 7.4) was used for extraction. PBS was added to the leaf discs at a ratio of 10 μL per mg of fresh weight leaf tissue. Cells were lysed on ice using a plastic pestle attached to a drill (Makita). The cell lysate was clarified by centrifugation at 4 °C and 10,600 g for 20 min. The supernatant was collected and immediately assayed for total soluble protein with the Bradford Assay. Supernatants were stored at −80 °C until required for ELISA and Western Blot analysis.

### 3.6. ELISA Analysis

ELISA was used to quantify the amount of plant-expressed CMG2-Fc. Microplate wells (Costar 3590) were coated with *Staphylococcus aureus* Protein A (Southern Biotech 7300-01S, Birmingham, AL) diluted at 50 μg/mL in PBS Buffer (pH 7.4) and incubated for 1 h at 37 °C. Blocking was achieved by a 15-min incubation with 5% nonfat dry milk (Safeway) diluted in PBS. All samples and controls were diluted in PBS and applied directly to the coated wells. For each microplate, a standard curve was generated with 5000; 1666.7; 555.6; 185.2; 61.7; 20.6; 6.9; and 2.3 ng/mL of purified CMG2-Fc (supplied by Planet Biotechnology Inc) diluted in PBS. Microplates were incubated with 50 μL of diluted samples and standards at 37 °C for 1 h. Goat anti-human IgG secondary antibody conjugated with horseradish peroxidase (Southern Biotech 2040-05, Birmingham, AL) diluted 1:3000 in PBS was added, and the microplates were incubated at 37 °C for 1 h. Detection was performed with 3,3′,5,5′-tetramethylbenzidine liquid substrate for ELISA. The reaction was stopped with ^1^N HCl and absorbance was measured at 450 nm. Each sample was assayed in triplicate, and CMG2-Fc concentrations were interpolated from the linear portion of the standard curve.

### 3.7. Bradford Protein Assay Analysis

The amount of total soluble protein present in the plant extracts was quantified using the Bradford Protein Assay [57]. Samples and controls diluted in PBS were applied directly to the microplate wells (Costar 3598). For each microplate, a standard curve was generated with 0.5, 0.4, 0.3, 0.2, 0.1, 0.05, and 0 mg/mL of bovine serum albumin diluted in PBS. After adding Bradford Protein Reagent (BioRad, Hercules, CA), the microplates were incubated at room temperature for 10 min, and absorbance was measured at 595 nm. Each sample was assayed in triplicate, and total soluble protein concentrations were interpolated from the standard curve.

### 3.8. Western Blot Analysis

Protein samples were denatured by heating for five min at 95 °C in sodium dodecyl sulfate (SDS) sample buffer and 1M dithiothreitol (DTT), for reduction of proteins at a final concentration of 90 mM DTT. Samples were fractionated with SDS-PAGE at 200 V for 30 min, and transferred to a 0.45 μm nitrocellulose membrane (GE Healthcare Biosciences, Pittsburgh, PA) at 120 V for 80 min. Blots were blocked with 5% nonfat dry milk diluted in PBS. Immunoblotting was performed using *Staphylococcus aureus* Protein A, followed by goat anti-human IgG secondary antibody conjugated with alkaline phosphatase (Southern Biotech 2040-04, Birmingham, AL). The blot was developed using the substrate BCIP/NBT (BioRad, Hercules, CA).

### 3.9. Statistical Analysis

Statistical analysis was performed using a two-sample *t*-test assuming unequal variances in Microsoft Excel.

## 4. Conclusions

In this study, we describe an efficient expression system for the transient production of recombinant proteins in plants. Furthermore, the usefulness of co-expressing gene silencing suppressors to overcome PTGS, which may be activated by the pathogenic effects of *Agrobacterium* over *N. benthamiana* or by the overexpression of the foreign CMG2-Fc protein, was demonstrated through qualitative and quantitative analysis. Suppressing the post-transcriptional gene silencing mechanism of *N. benthamiana* through the co-expression of certain suppressor proteins such as p1 and p25 effectively increased the expression level of a recombinant protein that was expressed transiently under the control of the CaMV 35S promoter.

High-level expression of CMG2-Fc was achieved using vacuum agroinfiltration of *N. benthamiana*, when using both intact plants and detached leaves. While the agroinfiltration of intact plants yielded higher CMG2-Fc levels than detached leaves, all expression levels were generally on the same order of magnitude. This result is significant, because a process relying on the latter approach is potentially more cost-effective to scale-up for large-scale protein production. The use of *N. benthamiana* detached leaves as biomass for expression eliminates the need for robotic manipulation of plants (harvested biomass can be transported via less expensive conveyor belts) as well as lighting systems for post-infiltration plant incubation; it also provides an opportunity for harvesting field grown plant tissues that would lower total capital investment and operating costs. Also, the reduced large scale cost of using detached leaves may offset the somewhat lower yields generated with this system.

The results of this project show the feasibility of producing a recombinant anthrax receptor fusion protein at high levels in plants, and provide further evidence that transient expression in *N. benthamiana* can be useful as a platform for the manufacturing of therapeutics. Future work will include evaluating the transient expression of CMG2-Fc under the control of a viral amplicon system [58]. It will be interesting to determine the effect that the co-expression of gene silencing suppressors will have on the viral amplicon driven expression of CMG2-Fc, compared to expression under the control of a constitutive promoter.

## Figures and Tables

**Table 1.** List of the different gene silencing suppressors screened for their effect on CMG2-Fc transient expression in *Nicotiana benthamiana*.

**PTGS Suppressor Protein**	**Plant Virus Source**	**Reference**
p1	Rice Yellow Mottle Virus	[27]
p10	Grapevine Virus A	[28]
p19	Tomato Bushy Stunt Virus	[29]
p21	Beet Yellow Virus	[30]
p24	Grapevine Leaf Roll Associated Virus	[31]
p25	Potato Virus X	[32]
p38	Turnip Crinkle Virus	[33]
2b	Cucumber Mosaic Virus	[34]
HcPro	Tobacco Etch Virus	[35]

**Table 2.** Conversion of expression values from ELISA to determine the amount of CMG2-Fc loaded on to each lane for Western Blot analysis.

**Lane**	**g CMG2-Fc/kg FW leaf ^a^**	**ng CMG2-Fc loaded on gel ^b^**
1	0.65	1300
2	0.05	100
3	0.39	780
4	0.08	160

a Data obtained from ELISA performed on individual samples;

b Conversion based on the use of 10 μL extraction buffer per mg of FW leaf tissue for sampling, and on the dilution of the sample aliquots prior to loading in the gel.
